# What theory, for whom and in which context? Reflections on the application of theory in the development and evaluation of complex population health interventions

**DOI:** 10.1016/j.ssmph.2016.12.005

**Published:** 2016-12-13

**Authors:** Graham F. Moore, Rhiannon E. Evans

**Affiliations:** Centre for the Development and Evaluation of Complex Interventions for Public Health Improvement (DECIPHer), School of Social Sciences, Cardiff University, Wales, United Kingdom

**Keywords:** Theory, Evaluation, Research methods, Public health

## Abstract

Recent years have seen a growing emphasis on the value of building and testing middle range theory throughout the development and evaluation of complex population health interventions. We agree that a coherent theoretical basis for intervention development, and use of evaluation to test key causal assumptions and build theory, are crucial. However, in this editorial, we argue that such recommendations have often been operationalised in somewhat simplistic terms with potentially perverse consequences, and that an uncritical assumption that an intervention explicitly based on theory is inherently superior carries significant risks. We first argue that the drive for theory-based approaches may have exacerbated a propensity to select ‘off-the-shelf’ theories, leading to the selection of inappropriate theories which distract attention from the mechanisms through which a problem is actually sustained. Second, we discuss a tendency toward over-reliance on individual-level theorising. Finally, we discuss the relatively slow progress of population health intervention research in attending to issues of context, and the ecological fit of interventions with the systems whose functioning they attempt to change. We argue that while researchers should consider a broad range of potential theoretical perspectives on a given population health problem, citing a popular off-the-shelf theory as having informed an intervention and its evaluation does not inherently make for better science. Before identifying or developing a theory of change, researchers should develop a clear understanding of how the problem under consideration is created and sustained in context. A broader conceptualisation of theory that reaches across disciplines is vital if theory is to enhance, rather than constrain, the contribution of intervention research. Finally, intervention researchers need to move away from viewing interventions as discrete packages of components which can be described in isolation from their contexts, and better understand the systems into which change is being introduced.

Recent years have seen a growing emphasis on the value of building and testing middle range theories (i.e. sets of empirically testable concepts which can be used to explain relationships and associations) throughout the development and evaluation of complex population health interventions. Guidance from the Medical Research Council (MRC) Population Health Research Network (PHSRN) for example, states that ‘best practice is to develop interventions systematically, using the best available evidence and appropriate theory’ (Craig et al., 2008). It also highlights the importance of building a ‘cumulative understanding of causal mechanisms’ so that we can learn from evaluations in order to ‘design more effective interventions and apply them appropriately across group and setting’ (Craig et al., 2008). Hence, theory is positioned as a crucial starting point for intervention development, while using evaluation to test and refine these theories is positioned as vital for maximising its contribution to a broader evidence base. Emphasis on theoretically-driven approaches has since continued to pervade evaluative thinking, with increased focus on integrating realist evaluation (Pawson & Tilley, 1997) principles into experimental designs (Bonell, Fletcher, Morton, Lorenc & Moore, 2012), new frameworks for intervention development such as 6 Steps in Quality Intervention Development (Wight, Wimbush, Jepson & Doi, 2015), Medical Research Council guidance on process evaluations (Moore et al., 2015), and supplementary tools to support integration of theory-based approaches with the MRC framework (De Silva et al., 2014).

We agree with the position advocated within all of these methodological works that a coherent theoretical basis for intervention development, and use of evaluation to test key causal assumptions and build theory, are crucial. Viewing evaluation not simply as a stop/go test of effectiveness, but as an opportunity to incrementally build understandings of what mechanisms work, and in what contexts (Jamal et al., 2015), compels us to be explicit regarding the causal assumptions driving an intervention and its evaluation, whether derived from formal social science theory, experience, common sense, or a combination of all of these various forms of ‘theory’ (Pawson & Tilley 1997).

However, in this editorial, we argue that such recommendations have often been operationalised in somewhat simplistic terms, with potentially perverse consequences, and that an uncritical assumption that an intervention explicitly based on theory is inherently superior carries significant risks. We first argue that the drive for theory-based approaches may have exacerbated a propensity to select ‘off-the-shelf’ theories, leading to the selection of inappropriate theories that distract attention from the mechanisms through which a problem is actually sustained. Second, we discuss a tendency toward over-reliance on individual-level theorising when the aim is to achieve community, organisational or population-level change. Finally, we discuss the relatively slow progress of population health intervention research in attending to issues of context, and the ecological fit of interventions with the systems whose functioning they attempt to change.

## The problem of ‘off the shelf’ theory

While all interventions are based on a theory (Pawson & Tilley 1997), whether implicit or explicit, ‘theory’ is often conceptualised narrowly as relating to middle-range theories from the social science literature. Adopting a well-established ‘off-the-shelf’ theory has been a common response among intervention researchers seeking to satisfy the assumption that theory-based interventions are inherently superior (Sniehotta, Presseau & Araújo-Soares, 2014). However, many formalised theories have in practice demonstrated limited utility in improving intervention effectiveness (Prestwich et al., 2014).

There are several potential explanations for this. First, the popularisation of a particular theory often appears to have little or nothing to do with its usefulness for enhancing intervention effectiveness. For example, the Stages of Change (Prochaska & DiClemente, 1983) model has driven much smoking cessation research and practice for the past 3 decades. However, as West has argued persuasively, this theory has largely acted as a security blanket for researchers and practitioners alike, providing false assurances regarding the likely effectiveness of efforts to promote cessation, despite growing evidence that its use does very little to make interventions more effective (West, 2005). The Theory of Planned Behaviour (TPB) has dominated health behaviour research for decades (Ajzen, 1985), though too is facing calls for its retirement (Sniehotta et al., 2014), given the growing evidence that its use has not tended to significantly enhance the effects of health behaviour interventions (Prestwich et al., 2014). The limited effect of many theory based interventions may also be due in part to the manner in which theories have been operationalized. In a review of the use of intervention theories to promote medication adherence for example, Munro, Lewin, Swart, and Volmink (2007) argue that while Social Cognitive Theory is one of the most commonly cited by intervention researchers, it's operationalisation is typically partial and inconsistent.

A fundamental problem with simply selecting a widely used theoretical framework is that viewing population health problems through such a narrow lens can serve to blinker attention away from important mechanisms which lie outside of that framework. One useful illustration of this problem is the Ontario Printed Educational Materials trial (Grimshaw et al., 2014, Presseau et al., 2016, Zwarenstein et al., 2016). The intervention used printed materials to influence physician behaviours including referral of diabetic patients to retinopathy, and prescription of thiazides. Variants of the intervention whose messaging was, or was not, informed by the TPB were equally ineffective compared to a no message control. The evaluation tested a hypothesis that materials would improve physicians’ behaviour, via improvements in key TPB constructs (i.e. attitudes, perceived norms and behavioural intentions). However, at baseline, physicians already had highly positive attitudes, normative perceptions and intentions toward the targeted behaviours, such that there was minimal scope for improvement in these mechanisms; guided by an inappropriate theory, the intervention targeted mechanisms which were not important in the aetiology of the problem, and failed to identify or address the mechanisms which were.

While we maintain that it is important to consider a broad range of theoretical perspectives in understanding and attempting to influence a population health problem, the tendency toward simply selecting a popular theoretical framework has arguably impeded progress in intervention science rather than accelerating it. A security blanket approach to adopting a popular theory may simply serve to provide false assurance that the causes of the problem are already fully understood, legitimising failures to fully engage with the problem and understand the most pertinent mechanisms driving it prior to intervening.

## The dominance of behavioural theory

The emphasis on the need to adopt theoretically-driven approaches has also led to, or at least reinforced, a reliance among intervention researchers on rather simplistic, individual-level theories (Moore et al., 2015). Indeed, practical instruction on theory integration often includes citation of a limited range of established psychological theories of behaviour change, such as Social Cognition Theory and Theory of Reasoned Action (Wight et al., 2015). Michie et al. (2013) work on developing a taxonomy of behaviour change techniques has gained much traction. However, while these works have made a substantial contribution to intervention research, they focus primarily, or exclusively, on psychological processes, and hence address the most proximal surface influences on behaviour. Hawe (2015) highlights a resultant tendency for many interventions to be minimally disruptive of the problems they seek to address; an imbalanced focus on the individual having encouraged a preoccupation with mechanisms that have minimal leverage, whilst rendering invisible those that are actually important to sustaining the problem. Salas (2015) for example, blames failures of the war on obesity in large part on its framing within an individualist paradigm which attempts to change society one individual (and one behaviour) at a time, ignoring structural contributors, whilst giving rise to iatrogenic effects through the legitimisation of weight related stigma. As Hawe (2015) argues, there is an ethical imperative to only commit resources to interventions where there is sound reason to believe that it targets mechanisms which have a realistic chance of bringing about change. Otherwise we risk directing scarce resource toward interventions which are negligible, or even negligent in their effects.

While the overly individualised nature of much dominant intervention theory is commonly acknowledged (Wight et al., 2015), it is rarer to see recognition that there is a wealth of alternative social science theory upon which intervention researchers could draw. Recent school-based interventions for example, such as INCLUSIVE (Bonell et al., 2014), have drawn upon complex and nuanced sociological theories of human functioning (Markham & Aveyard, 2003), that respond to the structural influences on many young people's health behaviours. In order to develop, evaluate and implement interventions that cause more than a minimal disruption in the problems they seek to address, it is vital to encourage more pluralistic approaches to the sources of theory that inform intervention. Guidance for population health researchers needs to move towards the inclusion of forms of theory that address deeper influences on behaviour, and away from the over-privileging of theory which addresses surface causes. As Hawe (2015) argues, more complex, system-level theories are often not as neatly packaged and ready to use as are more simplistic theoretical models, and hence there is substantial work to do to enhance the accessibility of such theoretical approaches (Brainard & Hunter, 2016).

## Incorporating context into theories of change

The vital importance of context in intervention research was perhaps most cogently articulated almost 20 years ago by Pawson and Tilley (1997), who argue that mechanisms of change are always contingent on context; what “works” in one time and place may be ineffective, or even harmful, elsewhere. However, population health science has to date been slower in responding to the importance of understanding context than it has in attending to mechanisms. MRC guidance for developing and evaluating complex interventions (Craig et al. 2008) for example made no mention of considerations of context when discussing intervention development, and only brief mention of the role of contextual factors in modifying intervention effects. Many of the aforementioned models of behavioural change are problematic in that they specify mechanisms linking actions to outcomes, but pay little attention to how those mechanisms function across time and space.

A clear illustration of the fundamental role of context in shaping how interventions work is evidenced by research on school smoking policies. Fifteen years ago, strong policies prohibiting smoking on school premises were associated with lower levels of youth smoking (Moore, Roberts & Tudor-Smith, 2001). This relatively simple intervention aimed to influence smoking through communicating strong norms around non-smoking behaviour, reflecting an implicit theory that addressing young people's perceptions of smoking as a normative behaviour could reduce their smoking risk. Hence, in that specific temporal context, this theorisation of the likely mechanisms of change appeared to be sound. However, replication with more recent data found a substantial weakening in these associations (Hallingberg et al, 2016).

In theorising the reasons for this shift in apparent effectiveness, we can consider the evolving context of tobacco use in this population. In 1998, when the data analysed by Moore et al. (2001) were collected, youth smoking had reached an all-time high and had become highly normalised. By 2013, smoking had become increasingly de-normalised through progressive legislation, with youth smoking reaching an all-time low. Within this much-changed macro-system, there is a need to revisit assumptions about the mechanisms through which youth smoking is sustained, and how it might be modified. Our assumptions about what will work in bringing about change, as well as our judgements on the relevance of past theory and evidence, must always consider the contingency of mechanisms across time and place, and be grounded in a contextually appropriate theory of how the problem is maintained. As Bonell et al. (2012) argue, a history of what has worked is precisely that, not a guarantee that the same intervention approaches will always work.

Emerging methodological work published by the National Institute for Health Research calls for researchers to attend more closely to issues of context (Howarth, Devers, Moore, O’Cathain & Dixon-Woods, 2016), embracing Hawe, Shiell and Riley (2009) view of interventions as disruptions to complex systems. From this perspective, interventions are fundamentally attempts to disrupt mechanisms which perpetuate and sustain a problem in a given time and place, and hence cannot be described, let alone understood, in isolation from the systems whose functioning they attempt to change. This represents something of a paradigm shift from traditional approaches which privilege detailed description of intervention components above an understanding of complexity arising from introducing something new into a system, but makes intuitive sense. Adding something new to a complicated system, like a car engine for example, would only be done in light of a good understanding of how the system (i.e. the engine) currently functions, and what difference the alteration will make to this. Other than through its interaction with the system, adding a new component has no absolute causal power to bring about change.

In population health, intervention researchers are typically interested in introducing change into complex rather than complicated systems (Glouberman & Zimmerman, 2002), and the consequences of interrupting the functioning of complex systems are usually highly unpredictable. Hence, viewing interventions in this way, our attention is drawn to the need for increased emphasis on understanding how a system in which a change is planned functions, before attempts are made to change it. While intervention research has traditionally privileged formal academic theories above local wisdom, as Berwick (2008) argues, those individuals involved in making changes in complex systems will often know more about mechanisms and contexts than third party evaluators can learn without engaging with them. Hence, co-producing interventions with stakeholders with intimate knowledge of the systems they attempt to alter represents an important means of ensuring congruence between theory and context.

## Conclusions: Future directions for intervention research

Following identification of limitations pertaining to current approaches to theory-driven intervention research, it is pertinent to consider recommendations for future research. Firstly, while researchers should consider a broad range of potential theoretical perspectives on a given population health problem, citing a popular off-the-shelf theory as having informed an intervention and its evaluation does not inherently make for better science. Indeed, in identifying, developing and justifying a theory of change to inform an intervention and its evaluation, researchers should demonstrate a clear understanding of how the problem under consideration is created and sustained in context. Secondly, a broader conceptualisation of theory that reaches across disciplines and moves beyond individual-level theorising is vital if theory is to enhance, rather than constrain, the contribution of intervention research. Thirdly, we need to move away from viewing interventions as discrete packages of components which can be described in isolation from their contexts, and better understand the systems into which we are attempting to introduce change before intervening. This will require us to continually privilege stakeholders in the development of complex interventions through intervention coproduction, ensuring that pertinent contextual influences can be sufficiently accommodated within theories of change, and that theories still retain their integrity in light of this context, or are abandoned and replaced by theories which do.
